# Elevated neurofilament light chain CSF/serum ratio indicates impaired CSF outflow in idiopathic intracranial hypertension

**DOI:** 10.1186/s12987-022-00403-2

**Published:** 2023-01-11

**Authors:** Sinah Engel, Johannes Halcour, Erik Ellwardt, Timo Uphaus, Falk Steffen, Frauke Zipp, Stefan Bittner, Felix Luessi

**Affiliations:** Department of Neurology, Focus Program Translational Neuroscience (FTN) and Immunotherapy (FZI), Rhine-Main Neuroscience Network (Rmn2), University Medical Center of the Johannes Gutenberg University Mainz, Langenbeckstrasse 1, 55131 Mainz, Germany

## Abstract

**Background:**

Impaired cerebrospinal fluid (CSF) homeostasis is central to the pathogenesis of idiopathic intracranial hypertension (IIH), although the precise mechanisms involved are still not completely understood. The aim of the current study was to assess the CSF/serum ratio of neurofilament light chain levels (QNfL) as a potential indicator of functional CSF outflow obstruction in IIH patients.

**Methods:**

NfL levels were measured by single molecule array in CSF and serum samples of 87 IIH patients and in three control groups, consisting of 52 multiple sclerosis (MS) patients with an acute relapse, 21 patients with an axonal polyneuropathy (PNP), and 41 neurologically healthy controls (HC). QNfL was calculated as the ratio of CSF and serum NfL levels. Similarly, we also assessed the CSF/serum ratio of glial fibrillary acidic protein (QGFAP) levels to validate the QNfL data. Routine CSF parameters including the CSF/serum albumin ratio (QAlb) were determined in all groups. Lumbar puncture opening pressure of IIH patients was measured by manometry.

**Results:**

CSF-NfL levels (r = 0.29, p = 0.008) and QNfL (0.40, p = 0.0009), but not serum NfL (S-NfL) levels, were associated with lumbar puncture opening pressure in IIH patients. CSF-NfL levels were increased in IIH patients, MS patients, and PNP patients, whereas sNfL levels were normal in IIH, but elevated in MS and PNP. Remarkably, QNfL (p < 0.0001) as well as QGFAP (p < 0.01) were only increased in IIH patients. QNfL was positively correlated with CSF-NfL levels (r = 0.51, p = 0.0012) and negatively correlated with S-NfL levels (r = − 0.51, p = 0.0012) in HC, while it was only positively associated with CSF-NfL levels in IIH patients (r = 0.71, p < 0.0001). An increase in blood-CSF barrier permeability assessed by QAlb did not lead to a decrease in QNfL in any cohort.

**Conclusions:**

The observed elevation of QNfL in IIH patients, which was associated with lumbar puncture opening pressure, indicates a reduced NfL transition from the CSF to serum compartment. This supports the hypothesis of a pressure-dependent CSF outflow obstruction to be critically involved in IIH pathogenesis.

**Supplementary Information:**

The online version contains supplementary material available at 10.1186/s12987-022-00403-2.

## Background

Idiopathic intracranial hypertension (IIH) is characterized by signs and symptoms of a raised intracranial pressure, including headache and impaired optic nerve function due to pressure-induced papilledema. It is typically seen in obese women of childbearing age with an incidence of 12–20 per 100,000 in contrast to an incidence of only around 0.5–2 per 100,000 within the general population. The management of IIH typically involves weight loss recommendation and medical treatment with acetazolamide or topiramate [1, 2]. In cases of rapid progression, surgical interventions including optic nerve sheath fenestration, dural venous sinus stenting and cerebrospinal fluid (CSF) diversion by shunt implantation can be considered [1].

The pathogenic mechanisms that cause IIH have not yet been fully elucidated, but impaired CSF homeostasis seems to play a major role [1]. Under physiological conditions, most CSF is formed in the cerebral ventricles, mainly via secretion by the choroid plexuses, although ependymal cells lining the ventricular system and brain parenchyma also contribute to its production and regulation. From the lateral ventricles, CSF flows through the left and right foramina of Monro to the third ventricle, from where it then passes to the fourth ventricle via the cerebral aqueduct of Sylvius. From the fourth ventricle, the CSF may exit through the foramen of Luschka laterally, or the foramen of Magendie medially to the subarachnoid space [3, 4].

Continuous exchange between the CSF and the interstitial fluid of the brain is essential for central nervous system (CNS) homeostasis as it enables the distribution of required compounds across CNS regions and contributes to the clearance of waste products [5]. One model of such a clearance system is the glia-associated lymphatic (glymphatic) system, which was described by Iliff and colleagues in 2012 [6]. It comprises the influx of subarachnoid CSF from perivascular spaces that surround penetrating arteries into the brain parenchyma along with consecutive convective bulk flow of interstitial fluid (ISF), which is followed by the clearance of CSF and brain metabolites along large-caliber draining veins. Expression of aquaporin-4 (AQP4) channels was proposed to facilitate CSF influx and to increase solute clearance in mice [5–7]. However, the concept of the glymphatic system has recently faced scrutiny (as summarized in [5]). Major points of criticism include the proposed role of AQP4 and that the high hydraulic resistance of the brain interstitium would greatly restrict such a convective flow. Moreover, transparenchymal flow of ISF, solutes and waste would appear difficult to reconcile with the required homeostasis of the brain microenvironment. Because of this, many experts in the field currently favor a model of diffusion within the brain parenchyma in combination with convection in the perivascular spaces to be the most likely mechanism of ISF-CSF exchange. Furthermore, diffusion between brain ISF and CSF occurring across the ependyma also largely contributes to the exchange [5, 7].

Similarly, the question of how and to what extent the CSF finally exits the brain and reaches the venous blood is also a subject of ongoing debate. CSF drainage occurs directly into the blood via arachnoid villi or granulations into the venous sinuses of the dura and into the lymphatic system via cranial and spinal nerves [5]. An additional pathway for CSF/ISF drainage appears to occur within dural lymphatic vessels to the cervical lymph nodes [8–11].

Based on these observations of physiological CSF circulation, Lenck et al. recently suggested a pathologic triad comprising the primary restriction of the venous CSF outflow pathway with secondary congestion of the glymphatic system and overflow of the lymphatic CSF outflow pathway to be responsible for the clinical manifestations of IIH [12].

Neurofilament light chains (NfL) are structural scaffolding proteins expressed by neurons of the CNS and the peripheral nervous system (PNS), which can be detected at elevated levels in CSF and blood following neuronal injury. The precise mechanisms by which NfL traffics between parenchymal, CSF and blood compartments are currently unknown, although several hypotheses have recently been reviewed by Gafson et al. [13]. Findings from previous experiments using tracer-labelled proteins injected intracerebrally suggest that perivenous draining might contribute to the egress of NfL from brain parenchyma [6]. Another possible exit route is the intramural periarterial drainage (IPAD) pathway [14], as it has been shown that soluble tracers injected into the ISF of the brain drain to cervical lymph nodes along the walls of cerebral arteries [15]. Of note, only 10–15% of tracer injected into cerebral hemispheres passed into the CSF, while the majority of the ISF passed to cervical lymph nodes via IPAD [15]. As of yet, there are no direct measurements of the proportion of NfL released from the brain that reaches the CSF. It is also not known whether any regional neuroanatomical selectivity is involved [13].

As the extent of NfL level increase often correlates with the severity of clinical symptoms, NfL is currently evaluated as a diagnostic, prognostic and treatment response biomarker in many neurological diseases [16–20]. A recent study suggested the prognostic potential of NfL in IIH patients since elevated CSF-NfL levels were observed to be associated with optic nerve damage reflected by pronounced papilledema, the development of visual field defects and atrophy of the retinal nerve fiber layer as measured by optical coherence tomography [21].

The strong correlation between NfL concentrations in CSF and serum in many neurological conditions [22–26] demonstrates that NfL must be able to exit the CSF compartment and enter the blood via currently unidentified mechanisms. Thus, we hypothesized that a restriction of the venous CSF outflow in IIH patients would be accompanied by an impaired transfer of NfL from CSF to serum reflected by an increase in the ratio of NfL levels in CSF to levels in serum (QNfL). To evaluate the validity of this hypothesis, the current study aims 1) to investigate the association of NfL in serum (S-NfL), CSF-NfL and QNfL values with lumbar puncture opening pressure in IIH patients, 2) to compare NfL measures of IIH patients with those of appropriate control groups, and 3) to validate QNfL findings by measuring an additional protein of CNS origin, glial fibrillary acidic protein (GFAP), in serum and CSF.

## Patients and methods

### Patient cohorts

Between 2016 and 2022, 87 patients with a diagnosis of IIH (Table 1), who presented at the Department of Neurology of the University Medical Center Mainz (Germany), were included in this cross-sectional study. In 67 of these patients with IIH we could acquire both serum and CSF samples at the same time point. We calculated QNfL using CSF-NfL divided by S-NfL. IIH was diagnosed according to the revised Friedman Criteria, requiring (a) papilledema, (b) normal neurologic examination except for abducens nerve palsy, (c) normal brain parenchyma in magnetic resonance imaging (MRI), (d) normal CSF composition, and (e) elevated lumbar puncture opening pressure at time point of diagnosis (≥ 25 cmH_2_O) [27]. The exclusion criteria were known history of neurological and/or autoimmune disease, head injury, or pregnancy. Paired serum and CSF samples were prospectively collected and stored in polypropylene tubes at − 80 °C. Lumbar puncture was performed in line with in-hospital standard operation procedures as part of the diagnostic routine and as therapeutic measure in case of risk for optic nerve damage until sufficient medical or surgical treatment was achieved. Lumbar puncture opening pressure was determined using a manometer with the patient lying in supine position. Information on the presence of headache and visual disturbances, medication, weight and height were recorded. An ophthalmologist assessed visual acuity and visual fields using automated perimetry; transorbital sonography was performed by a trained investigator following previously published protocols immediately prior to lumbar puncture [28].

**Table 1:** Baseline characteristics of IIH patients

	All patients (n = 87)
Sex, n (%)	
Male	12 (13.8%)
Female	75 (86.2%)
Age at sample collection (years), median (IQR)	30.0 (25.0–40.0)
Disease duration at sample collection (months), median (IQR)	0 (0–4.0)
Weight at sample collection (kg), mean ± SD	104.2 ± 26.5
Body mass index (kg/m^2^), mean ± SD	37.1 ± 9.8
Cardiovascular risk factors other than obesity n (%)	
Yes	16 (18.4%)
No	71 (81.6%)
Lumbar puncture opening pressure at sample collection (cmH_2_O), mean ± SD	34.0 ± 9.6
IIH medication at sample collection, n (%)	
None	58 (66.7%)
Acetazolamide monotherapy	23 (26.4%)
Acetazolamide + topiramate/furosemide	6 (6.9%)
Headache, n (%)	
Yes	70 (80.5%)
No	17 (19.5%)
Impaired visual acuity, n (%)	
Yes	50 (57.5%)
No	37 (42.5%)
Visual field defects, n (%)	
Yes	21 (24.1)
No	66 (75.9)
Diplopia, n (%)	
Yes	7 (8.0%)
No	80 (92.0%)
Optic nerve sheath diameter (cm), mean ± SD, n = 63	0.66 ± 0.07

As control groups, we included 52 patients with relapsing-remitting multiple sclerosis (RRMS) or clinically isolated syndrome (CIS) according to the 2017 revised version of the McDonald diagnostic criteria [29], who underwent lumbar puncture as part of the diagnostic workup at time point of initial diagnosis. All of the MS patients had a relapse within 3 months prior to sample collection, but none were treated with steroids or disease-modifying treatment before sample collection. Furthermore, 41 age- and sex-matched neurologically healthy controls (HC), who underwent lumbar puncture in order to exclude organic causes of unspecific symptoms such as vertigo, paresthesia and headache, were also included. All of the HC patients were free from focal neurological deficits in clinical neurological examination, had normal brain MRI scans and normal CSF composition. As a third control cohort, we included 21 patients with axonal non-inflammatory polyneuropathy (PNP), where the source of elevated S-NfL levels should be in the peripheral nervous system.

### CSF analyses

CSF analyses were performed in a standardized fashion as part of the routine diagnostic workup. CSF samples with artificial blood contamination (defined as erythrocyte count  > 100/µl) were excluded from the analyses. CSF concentrations of albumin were determined with immunonephelometry; quotients of albumin (QAlb) were defined as the ratio of CSF concentration to the corresponding serum concentration.

### Single molecule array analysis of NfL and GFAP levels

NfL and GFAP levels were determined using the highly sensitive single molecule array (SiMoA) technology. Samples were measured in duplicates in several rounds by SiMoA HD-1 (Quanterix, USA) using the NF-Light Advantage and GFAP Discovery kits according to manufacturer’s instructions. Resorufin-β-d-galactopyranoside (RGP) was incubated at 33 °C for 60 min prior to running the assay. The coefficient of variation (CV, as a percentage) of the two replicates was obtained by dividing the standard deviation of both replicates by the mean of both replicates multiplied by 100. CVs above 20% (or missing replicate result) were measured twice. Finally, the mean intra-assay CV of 7.8% was obtained by averaging all individual sample CVs (serum and CSF, NfL and GFAP). Two low and high controls, consisting of recombinant human antigen, were included in each sample run to monitor plate-to-plate variation, which was less than 10% overall for both assays (NfL low: mean 3.0 pg/ml, inter-assay CV 6.0%; NfL high: mean 147.2 pg/ml, inter-assay CV 4.7%; GFAP low: mean 15.11 pg/ml, inter-assay CV 6.6%; GFAP high: mean 1056.4 pg/ml, inter-assay CV 8.8%). Measurements were performed in a blinded fashion without information about clinical data.

### Z-score analysis for S-NfL levels

To overcome the notion that S-NfL levels positively correlate with age and negatively correlate with body mass index (BMI), we calculated z-scores using a reference database of 10,133 blood samples from 5390 healthy individuals [30], correcting S-NfL levels for BMI and age.

### Statistical analyses

All statistical analyses were performed using GraphPad (version 9.3.1) or SPSS (version 28.0). Continuous variables were tested for normal distribution using the Shapiro-Wilk normality test. In case of normal distribution, continuous variables are depicted as mean ± standard deviation (SD), whereas non-normally distributed variables are referred to by their median and interquartile range (IQR). Categorical variables are described by numbers and percentages. Associations between two continuous variables were assessed using Spearman’s correlation analysis. Comparisons of the mean between two continuous variables were tested by Student’s t-test in case of normal distribution and by Mann-Whitney U test in non-normally distributed variables. In case of more than two continuous variables, comparisons of the mean were performed using Kruskal-Wallis test with post hoc pairwise comparisons using Wilcoxon rank sum exact test with Bonferroni correction. If not stated otherwise, we used Benjamini-Hochberg correction with a false discovery rate of 0.1 to account for multiple testing.

## Results

### CSF-NfL and QNfL are associated with increased lumbar puncture opening pressure in IIH patients

Descriptive statistics of the IIH cohort are presented in Table 1. In line with the prevalence of the general patient population, the majority of patients included in this study were female (86.2%). The mean BMI was clearly elevated (37.1 kg/m^2^) and most of the patients presented with headache or visual impairment as first symptoms.

**Fig. 1:**
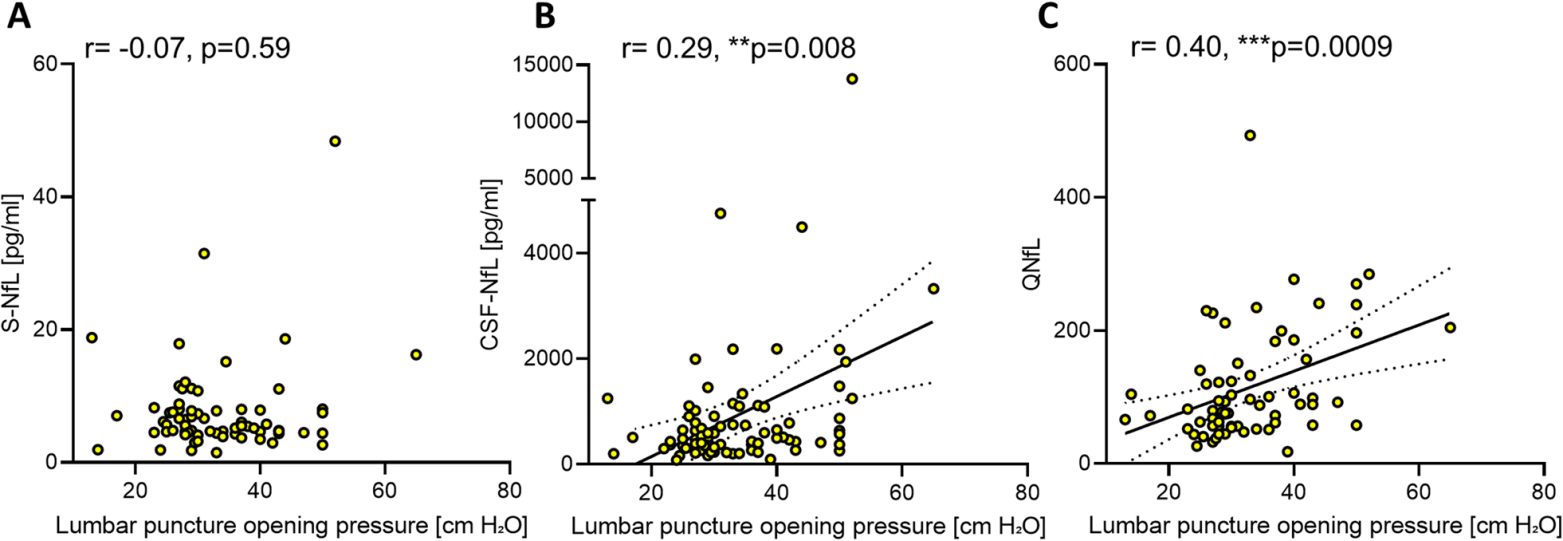
Only CSF-NfL and QNfL are positively correlated with lumbar puncture opening pressure in IIH patients. Lumbar puncture opening pressure (displayed in cmH_2_O) was not associated with S-NfL levels (**A**), but positively correlated with CSF-NfL levels (**B**). The calculated CSF/serum ratio of NfL (QNfL) showed a positive correlation with lumbar puncture opening pressure (**C**) Associations are illustrated by scatter plots with linear regression lines and 95% confidence bands. *CSF-NfL* cerebrospinal fluid neurofilament light chain, *QNfL* CSF/serum ratio of neurofilament light chain, *S-NfL* serum neurofilament light chain

CSF-NfL and S-NfL levels demonstrated a moderate positive correlation (r = 0.59, p < 0.0001). Interestingly, only S-NfL levels were positively correlated with age at sampling (r = 0.51, p < 0.0001), whereas CSF-NfL levels showed no association with age (r = 0.24, p = 0.051). Furthermore, S-NfL levels were unrelated to the lumbar puncture opening pressure at time point of sample collection (r = − 0.07, p = 0.59; Fig. 1A), while CSF-NfL levels were positively correlated with the lumbar puncture opening pressure (r = 0.29, p = 0.008; Fig. 1B).

In reference to the concept of the CSF/serum ratio of albumin as a marker of blood-CSF barrier permeability [31], we next calculated the ratio of CSF-NfL and S-NfL concentrations as a marker of functional CSF-blood permeability (QNfL). Notably, the association of lumbar puncture opening pressure with QNfL was more pronounced than the correlation with CSF-NfL levels (0.40, p = 0.0009; Fig. 1C). The correlation between QNfL and CSF-NfL was high (r = 0.71, p < 0.0001, Additional file 1: Fig. S1A), whereas QNfL was not associated with S-NfL (r = − 0.05, p = 0.68, Additional file 1: Fig. S1B and Additional file 1: Table S1).

### QNfL is only increased in IIH patients

Our findings demonstrate that higher lumbar puncture opening pressure is associated with an imbalance of NfL concentrations between the CSF and serum compartments in IIH patients. In order to further investigate whether this imbalance emanates from an increased central NfL release or from an impairment of CSF-to-serum transfer, we next compared CSF-NfL, S-NfL and QNfL of IIH patients with several control groups. Patients without a neurological disease served as a reference for normal findings (HC). Furthermore, we looked at recently diagnosed and still treatment naïve MS patients with an acute relapse and patients with an axonal polyneuropathy (PNP) as two exemplary diseases with a central and peripheral source of NfL, respectively. Characteristics of the MS and PNP patients are depicted in Additional file 1: Table S2.

**Fig. 2:**
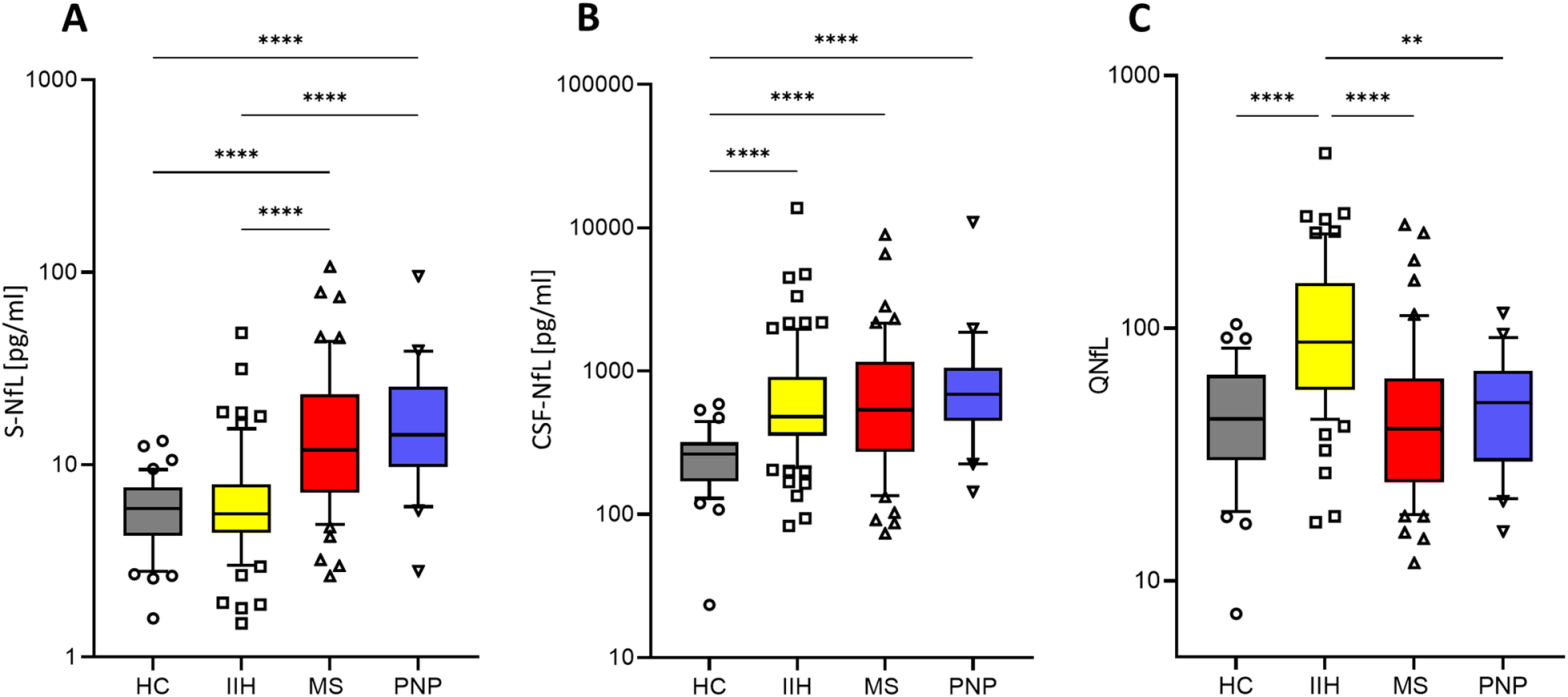
QNfL levels are elevated in IIH patients only. S-NfL levels were increased in MS patients and PNP patients (**A**), whereas CSF-NfL levels were increased in all patient groups, compared to the non-neurological controls (HC) (**B**). The CSF/serum ratio of NfL (QNfL) was only increased in IIH patients (**C**). Horizontal lines of the boxplots denote the median; boxes extend from the 25th to 75th percentile, whiskers from 10 to 90th percentile; individual data points are below the 10th or above the 90th percentile; **p < 0.01, ****p < 0.0001. *CSF-NfL* cerebrospinal fluid neurofilament light chain, *HC* healthy controls, *IIH* idiopathic intracranial hypertension, *PNP* polyneuropathy, *MS* multiple sclerosis, *QNfL* CSF/serum ratio of neurofilament light chain, *S-NfL* serum neurofilament light chain

S-NfL levels of IIH patients did not differ from those of HC, whereas the MS and PNP patients had increased S-NfL levels (χ^2^(3) = 51.9, p < 0.0001, Fig. 2A and Table 2). Calculated z-scores of S-NfL levels, correcting for age and BMI, showed a similar tendency with significantly higher values in MS and PNP and most importantly no significant difference between HC and IIH (Table 2 and Additional file 1: Fig. S1). On the other hand, CSF-NfL levels of IIH patients were comparable to those of the MS and PNP patients and significantly higher than in HC (p < 0.0001, Fig. 2B and Table 2). Of note, QNfL, the ratio between CSF-NfL levels and S-NfL levels, was only increased in patients with IIH compared to all other groups (χ^2^(3) = 41.8, p < 0.0001, Fig. 2C and Table 2). This finding holds also true after adjusting QNfL values for BMI as a covariate.

**Table 2:** Comparison of IIH patients with healthy controls, MS patients and PNP patients

	HC, n = 41	IIH, n = 87	MS, n = 52	PNP, n = 21	Group comparisons
QNfL, median (IQR)	43.7 (30.0–65.3)	87.9 (56.9–151.0)	40.0 (24.5–63.3)	50.6 (29.7–67.6)	p < 0.0001 IIH > HC, ON, MS
S-NfL (pg/ml), median (IQR)	5.9 (4.3–7.6)	5.7 (4.5–8.0)	12.0 (7.2–23.3)	14.3 (9.8–25.5)	p < 0.0001 MS and PNP > HC, IIH
CSF-NfL (pg/ml), median (IQR)	263.8 (170.2–318.6)	480.2 (354.9–909.4)	536.3 (273.5–1161)	691.7 (450.8–1058)	p < 0.0001 IIH, MS, PNP > HC
Z-score S-NfL, median (IQR)	0.3 (-0.6–1.1)	0.9 (0–1.6)	1.9 (0.9–2.9)	1.3 (0.3–2.4)	p < 0.0001 MS > HC, IIH; PNP > HC
Age at time point NfL (years), median (IQR)	29 (25.5–37)	30 (25.0–40.0)	30 (24–36.8)	55 (47.0–61.0)	p < 0.0001 PNP > HC, IIH, MS
BMI (kg/m^2^), mean ± SD	26.4 ± 7.8	37.1 ± 9.8	25.7 ± 6.3	26.8 ± 4.7	p < 0.0001 IIH > HC, MS, PNP
QAlb*10^3^, median (IQR)	4.2 (3.2–5.2)	4.2 (3.0–5.6)	4.5 (3.7–6.3)	5.7 (4.5–11.1)	P = 0.0013 PNP > HC, IIH
S-Alb (g/l),median (IQR)	43.5 (41.6–45.2)	41.6 (39–44.5)	45.8 (43.5–48.1)	42.8 (40.7–44.7)	p < 0.0001 MS > HC, IIH, PNP
CSF-Alb (mg/l), median (IQR)	180 (140–237.5)	175 (128–235)	216 (163.5–276)	242 (169.5–471.5)	P = 0.0003 PNP > HC, IIH; MS > IIH
QIgG*10^3^, median (IQR)	2.0 (1.7–2.6)	2.1 (1.5–3.0)	4.0 (3.1–5.3)	2.8 (2.1–5.3)	p < 0.0001 MS > HC, IIH PNP > IIH

*CSF-NfL* cerebrospinal fluid neurofilament light chain, *HC* healthy controls, *IIH* idiopathic intracranial hypertension, *IQR* interquartile range, *MS* multiple sclerosis, *n.s.* not significant, *PNP* Polyneuropathy, *QAlb* albumin ratio, *S-Alb* serum albumin, *CSF-Alb* cerebrospinal fluid albumin, *QIgG* immunoglobulin G ratio, *QNfL* neurofilament light chain ratio, *S-NfL* serum neurofilament light chain

In the neurologically healthy controls, S-NfL and CSF-NfL levels showed a positive association (r = 0.38, p = 0.02, Additional file 1: Table S2). QNfL was positively correlated with CSF-NfL levels and negatively correlated with S-NfL levels (Additional file 1: Table S2). The association of S-NfL and CSF-NfL in the acute MS group was high and QNfL was only positively associated with CSF-NfL levels, whereas S-NfL levels did not show an association with QNfL (Additional file 1: Table S2), a finding which was also seen in PNP patients. In IIH patients, QNfl was only correlated with CSF-NfL, but not with S-NfL (Additional file 1: Table S2 and Additional file 1: Fig. S2).

### Increase in blood-CSF barrier permeability is not associated with decrease in QNfL

As QAlb is a marker of blood-CSF barrier permeability, we next investigated the association between CSF-NfL, S-NfL and QNfL with QAlb. In IIH patients, we could not find any significant correlation between QAlb and S-NfL, CSF-NfL or QNfL (see Additional file 1: Table S3). Similarly, there were no associations in HC or PNP patients, whereas in MS patients, we observed a positive correlation between QAlb and QNfL. Most importantly, a decrease in blood-brain barrier integrity (reflected by an increase in QAlb) did not lead to a decrease in QNfL.

### CSF-GFAP levels and QGFAP are increased in IIH patients

**Fig. 3:**
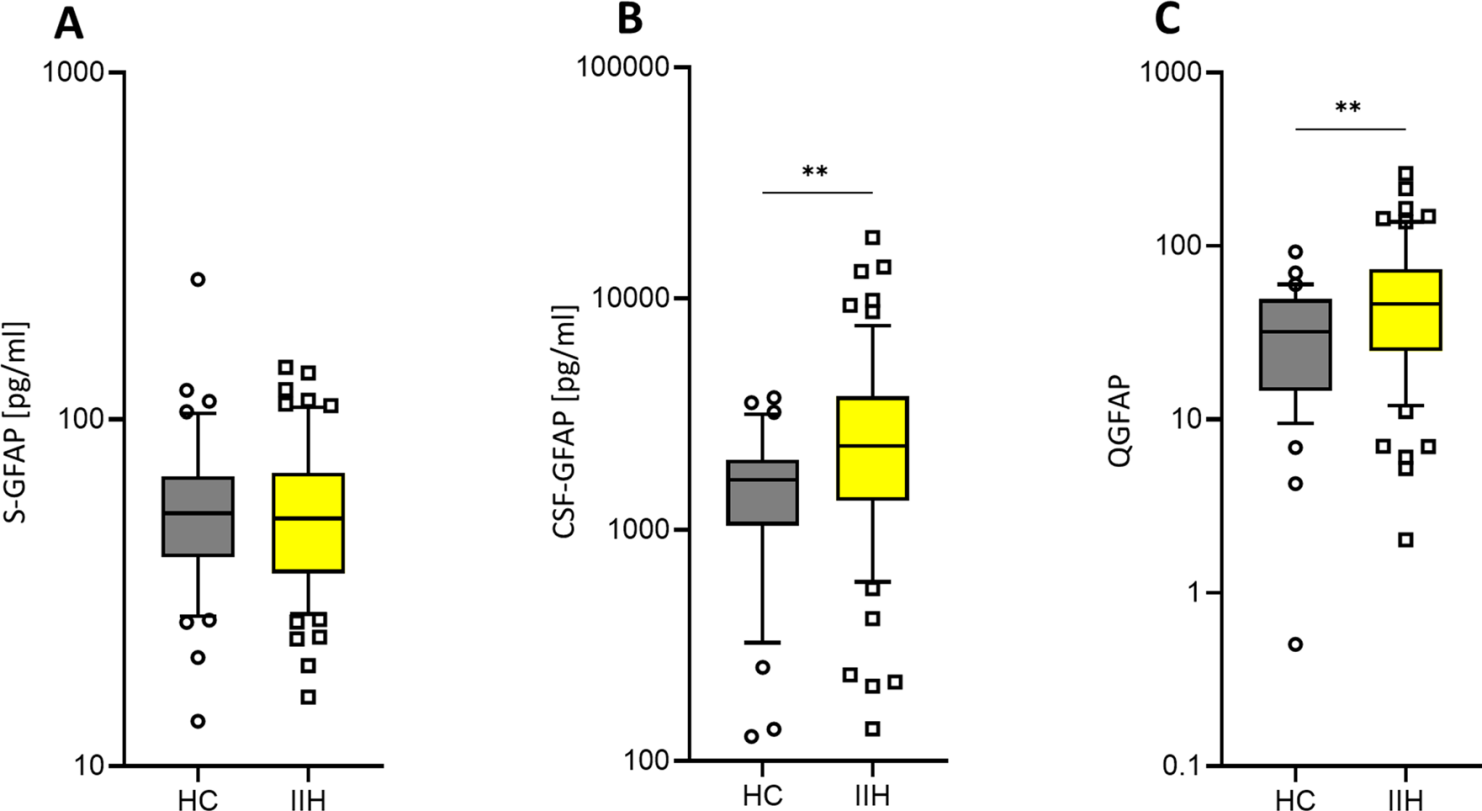
QGFAP levels are also elevated in IIH. While serum GFAP (S-GFAP) levels of IIH patients did not differ from the non-neurological controls (HC, **A**), IIH patients had increased CSF-GFAP (**B**) and QGFAP levels (**C**). Horizontal lines of the boxplots denote the median; boxes extend from the 25th to 75th percentile, whiskers from 10 to 90th percentile; individual data points are below the 10th or above the 90th percentile; **p < 0.01. *CSF-GFAP* cerebrospinal fluid glial fibrillary acidic protein, *HC* healthy controls, *IIH* idiopathic intracranial hypertension, *QGFAP* CSF/serum ratio of glial fibrillary acidic protein, *S-GFAP* serum glial fibrillary acidic protein

We also assessed GFAP concentrations (a protein mainly expressed by CNS astrocytes) in serum and CSF of IIH patients and neurologically healthy controls, and calculated the respective CSF-GFAP/S-GFAP ratio (QGFAP). The comparison of GFAP parameters between IIH patients and HCs showed similar trends as for NfL, although the differences were less pronounced. S-GFAP levels did not differ between IIH patients and HCs (median and IQR, 51.9 (36.0–70.2) vs. 53.7 (40.1–68.6), p = 0.72, Mann-Whitney test, Fig. 3A), whereas CSF-GFAP levels (2306 pg/ml (1339–3770) vs. 1648 pg/ml (1042–2015), p = 0.0028, Fig. 3B) and QGFAP were higher in IIH patients than in HC (46.3 (24.6–73.3) vs. 32.0 (14.6–49.3), p = 0.009, Fig. 3C). However, neither CSF-GFAP levels (r = 0.02, p = 0.90) nor QGFAP (r = 0.14, p = 0.27) showed an association with the lumbar puncture opening pressure in IIH patients.

## Discussion

It is widely accepted that IIH is associated with a disturbance of CSF homeostasis, although the precise mechanisms involved are largely unknown. A pathologic cascade originating from an impairment of CSF outflow into the venous system has been suggested to play a crucial role in its pathogenesis [1]. The here presented observational study offers clinical evidence in support of the hypothesis of a reduced permeability between CSF and blood compartments in IIH by demonstrating an elevation of the NfL CSF/serum ratio in IIH patients that was also associated with lumbar puncture opening pressure as a measure of disease severity.

The comparison of CSF-NfL, S-NfL, and QNfL levels in IIH patients with a number of control groups revealed several interesting findings (Table 3).

**Table 3:** Overview of NfL measures of patient groups in comparison to healthy controls

	**CSF-NfL**	**S-NfL**	**QNfL**
HC	normal	normal	normal
IIH	↑	=	↑
MS	↑	↑	=
PNP	↑	↑	=

Overview of NfL measure alterations of IIH, MS patients and PNP patients in comparison to the values measured in our healthy control group, where ↑ relates to increased values and = means that there was no difference

*CSF-NfL* cerebrospinal fluid neurofilament light chain, *HC* healthy controls, *IIH* idiopathic intracranial hypertension, *MS* multiple sclerosis, *PNP* Polyneuropathy, *QNfL* neurofilament light chain ratio, *S-NfL* serum neurofilament light chain

First, we found that CSF-NfL levels of IIH patients were higher than in neurologically healthy controls and did not differ from MS patients with an acute relapse. A previous study on NfL in IIH described higher CSF-NfL levels in IIH patients with moderate to severe papilledema when compared to IIH patients with mild or no papilledema. Furthermore, the thickness of the retinal nerve fiber layer measured by optical coherence tomography was inversely correlated with CSF-NfL concentrations. Therefore, the authors suggested that the increased intracranial pressure resulted in axonal damage of the optic nerves reflected by a release of NfL to the CSF [21]. However, we hypothesize that NfL is also retained in the CSF due to a functional CSF outflow obstruction, which leads to high CSF-NfL levels even in the case of only minimal (or no) neuronal damage.

Second, S-NfL levels as well as z-scores of S-NfL [30] of our IIH patients lay within normal ranges and showed no differences in comparison to the non-neurological controls, which is in line with the study of Beier et al. that observed normal S-NfL levels in IIH patients as well [21]. This shows that, in contrast to other neurological diseases [17, 18, 25], S-NfL is not a suitable biomarker to diagnose IIH.

Third, the CSF/serum ratio of NfL was markedly increased in IIH patients, whereas we observed no differences in QNfL values between the other groups. Remarkably, in MS patients with an acute relapse, QNfL values were normal. We considered two possible explanations for the observed differences in QNfL between MS and IIH patients: (1) increase in NfL transfer from CSF to serum in MS, or (2) decrease in NfL transfer from CSF to serum in IIH. Notably, the median CSF/serum ratio of albumin (QAlb), which is a marker of blood-CSF barrier integrity [31], was within normal ranges in these groups and showed no significant differences between patients with IIH and MS. In contrast to these groups, axonal PNP patients had slightly higher QAlb values, which may be related to the mild blood-brain barrier disruption that is observed regularly in this patient group [32]. Moreover, the CSF/serum ratio of NfL was positively correlated with the CSF/serum ratio of albumin in MS patients, whereas one would expect a negative association in the case of an enhanced NfL transfer in the presence of an increased blood-CSF barrier permeability. Therefore, it seems unlikely that an increased NfL leakage caused by impaired blood-CSF barrier integrity of MS patients was the underlying reason for the differing QNfL values in MS and IIH patients.

It should be noted that NfL is not only expressed by neurons of the CNS, but also by neurons of the PNS [33]. Therefore, peripheral NfL release into the blood could potentially impact its CSF/serum ratio, which has been investigated by a study in patients with Guillain-Barré Syndrome (GBS). Here, the authors observed a strong association between S-NfL levels and QNfL, whereas CSF-NfL levels were unrelated to QNfL, which led them to assume that PNS damage was more pronounced than CNS damage in their cohort of GBS patients [34]. When comparing QNfL values derived from a recent study comparing patients with GBS, chronic inflammatory demyelinating PNP, and non-inflammatory PNP with healthy controls, we did not observe any differences in QNfL between the groups [35] (Additional file 1: Table S4). In line with the latter finding, the cohort of axonal PNP patients in our study also showed QNfL values comparable to HC.

The findings of our current study support the assumption that QNfL can help to reveal the origin of NfL: in the non-neurological controls, QNfL was positively correlated with CSF-NfL levels and negatively correlated with S-NfL levels with similar correlation coefficients, which indicates that central and peripheral NfL release are balanced. In contrast, QNfL was only positively associated with CSF-NfL levels in MS patients, but unrelated to S-NfL levels, thus highlighting the evident central source of NfL release in these patients. In our cohort of IIH patients, QNfL also showed a positive correlation with CSF-NfL levels, but not with S-NfL levels, which underlines the imbalance between CSF and S-NfL levels with an excess of centrally localized NfL in this patient group. Viewed in isolation, this either could be due to an NfL release by neurons of the CNS or caused by an impaired transfer of NfL from CSF to serum. However, in consideration of all the current observations and recent publications discussed above, we think it is most likely attributable to a combination of both processes, i.e., a release of NfL to the CSF caused by damage of central neuronal structures and an impairment of CSF outflow into the venous blood, mirrored by the elevated QNfL in our IIH cohort.

The eponymous characteristic of IIH is a rise in intracranial pressure (ICP), which, in clinical practice, is usually assessed by repeated measurements of the lumbar puncture opening pressure. Whether the observed elevated ICP is rather due to CSF hypersecretion or outflow obstruction is still under debate. Of note, tumors accounting for an elevated CSF production or conditions leading to a secondary outflow obstruction (e.g., meningitis) have to be excluded before diagnosing IIH [1, 36]. In the current study, we observed a positive correlation of CSF-NfL levels with lumbar puncture opening pressure at the time point of NfL sample collection, which was not detectable for S-NfL levels. This is in line with findings of an earlier study [21]. Remarkably, the association of lumbar puncture opening pressure with QNfL was even more pronounced than its association with CSF-NfL. This supports the hypothesis of CSF outflow obstruction as a major driver in IIH pathogenesis and indicates that the higher the intracranial pressure the more pronounced the impairment of CSF outflow. In contrast, the isolated hypersecretion of CSF without accompanying outflow obstruction would rather lead to lower CSF-NfL concentrations due to dilution effects potentially in combination with enhanced clearance from the CNS compartment. With regard to QNfL, such a scenario of hypersecretion would thus not be expected to lead to an increase but rather to stable or even decreased QNfL values.

QNfL might serve as a biomarker of disease severity in IIH, which is of special interest as the measurement of lumbar puncture opening pressure is time-consuming and interference-prone. However, although the positive correlation between QNfL and lumbar puncture opening pressure is significant, the strength of correlation is modest and there is a substantial scatter about the regression line. This suggests that additional, so far unknown, factors likely also influence the observed association. A prospective study including longitudinal measurements in a deeply characterized cohort that allows correction for potential confounding factors should be performed to improve the prognostic accuracy of QNfL. The next step would then be the evaluation of the suitability of QNfL to inform treatment decisions.

In order to further evaluate our findings, we additionally assessed GFAP concentrations in IIH patients and the non-neurological controls. The measurement of GFAP was chosen since previous histopathological studies have reported the presence of patchy astrogliosis in IIH patients [37], and due to its almost exclusive expression by CNS astrocytes [38]. Similarly to NfL, we observed increased CSF-GFAP and QGFAP values in IIH patients, whereas S-GFAP levels did not differ from those of the non-neurological controls. However, none of the GFAP parameters were associated with the lumbar puncture opening pressure in IIH patients.

Taken together, our main findings comprise that lumbar puncture opening pressure of IIH patients shows the strongest association with the CSF/serum ratio of NfL and that this ratio is increased in IIH patients in comparison to our control groups. Moreover, the GFAP ratio was also elevated in IIH. These observations suggest an impaired clearance of brain tissue metabolites to the periphery in IIH patients, which is positively associated with lumbar puncture opening pressure elevation. This supports the recently evolving hypothesis concerning IIH pathogenesis comprising a pressure-dependent CSF outflow obstruction with secondary congestion of the glymphatic system [8, 12].

Major strengths of our study are the inclusion of several control groups and the additional assessment of GFAP levels that allowed us to set the NfL measures into clinical context. However, our findings are limited by the comparatively small sample size and by the cross-sectional nature of our current data. Our observations are purely correlative and do not address the pathological mechanisms involved. Specifically, it is currently unknown how NfL is released into the CSF and blood. Recently, a study using a quantitative immunoprecipitation-mass spectrometry assay reported three major NfL fragments in CSF, whereas no full-length NfL was identified in CSF [39]. Thus, our study is limited by the ability of the NfL SiMoA assay to detect all the key NfL fragments in CSF and serum. These points need to be addressed in future studies in model systems using different quantification methods of NfL fragments for validation.

In addition, our PNP cohort was significantly older than the other three groups, limiting the comparability in this study. We tried to overcome this problem by also calculating the z-scores for S-NfL. To evaluate the potential of QNfL as an additional or alternative disease severity biomarker in IIH, future studies are needed to evaluate the longitudinal alterations of NfL parameters and their development upon normalization of lumbar puncture opening pressure in sequential follow-up measurements of IIH patients.

## Conclusions

This study demonstrates that S-NfL is not a suitable biomarker to identify patients with IIH during the initial diagnostic work-up, but that CSF-NfL levels and especially the CSF/serum ratio of NfL correspond well with lumbar puncture opening pressure. Moreover, our findings indicate an impairment of NfL transfer from the CSF to serum compartment in IIH patients, which supports the hypothesis of a restricted CSF outflow with potential secondary congestion of the glymphatic system being critically involved in IIH pathogenesis.

## Supplementary Information

**Additional file 1: Figure S1.** Z-Scores for S-NfL, correcting for age and body mass index (BMI). **Figure S2.** Increased QNfL in IIH is associated with elevated CSF-NfL. **Table S1.** Spearman correlation of QNfL with S-NfL and CSF-NfL. **Table S2.** Characteristics of MS and PNP patients. **Table S3.** Spearman correlation of QAlb with S-NfL, CSF-NfL and QNfL. **Table S4.** QNfL derived from previously published CSF-NfL an P-NfL in patients with inflammatory polyneuropathies and controls (34).

## Data Availability

The datasets used and/or analysed during the current study are available from the corresponding author on reasonable request.
