# Supplementary material for: Elevated neurofilament light chain CSF/serum ratio indicates impaired CSF outflow in idiopathic intracranial hypertension
Source: Fluids Barriers CNS. 2023 Jan 11;20:3. doi: 10.1186/s12987-022-00403-2 (PMC9832777; doi:10.1186/s12987-022-00403-2)
Supplement: Supplementary file 1 — Additional file 1: Figure S1. Z-Scores for S-NfL, correcting for age and body mass index (BMI). Figure S2. Increased QNfL in IIH is associated with elevated CSF-NfL. Table S1. Spearman correlation of QNfL with S-NfL and CSF-NfL. Table S2. Characteristics of MS and PNP patients. Table S3. Spearman correlation of QAlb with S-NfL, CSF-NfL and QNfL. Table S4. QNfL derived from previously published CSF-NfL an P-NfL in patients with inflammatory polyneuropathies and controls (34). [file 12987_2022_403_MOESM1_ESM.docx]

**Suppl. Fig. 1**

**Z-Scores for S-NfL, correcting for age and body mass index (BMI).**

Horizontal lines of the boxplots denote the median; boxes extend from the 25th to 75th percentile, whiskers from 10th to 90th percentile; individual data points are below the 10th or above the 90th percentile; *p < 0.05, ***p < 0.001, ****p < 0.0001. Abbreviations: HC: healthy controls, IIH: idiopathic intracranial hypertension, MS: multiple sclerosis, PNP: polyneuropathy

**Suppl. Fig. 2**

**A**

**B**

r= 0.71, ****p<0.0001

r= -0.05, p=0.68

**Increased QNfL in IIH is associated with elevated CSF-NfL.**

In patients with IIH, (**A**) CSF-NfL levels, but not (**B**) S-NfL levels correlate positively with QNfL. Associations are illustrated by scatter plots with linear regression lines and 95% confidence bands.

Abbreviations: CSF-NfL: cerebrospinal fluid neurofilament light chain, QNfL: CSF/serum ratio of neurofilament light chain, S-NfL: serum neurofilament light chain.

## Supplementary Table 1: Spearman correlation of QNfL with S-NfL and CSF-NfL

|  | **HC, n=41** | **IIH, n=67** | **MS, n=52** | **PNP, n=21** |
| --- | --- | --- | --- | --- |
| QNfL vs. S-NfL | r=-0.51  **p=0.0012 | r=-0.05  p=0.68 | r=-0.05  p=0.75 | r=-0.25  p=0.28 |
| QNfL vs. CSF-NfL | r=0.51  **p=0.0012 | r=0.71  ****p<0.0001 | r=0.63  ****p<0.0001 | r=0.44  *p=0.04 |
| S-NfL vs. CSF-NfL | r=0.38  *p=0.02 | r=0.59  ****p<0.0001 | r=0.69  ****p<0.0001 | r=0.70  ***p=0.0004 |

Abbreviations: CSF-NfL: cerebrospinal fluid neurofilament light chain, HC: healthy controls, IIH: idiopathic intracranial hypertension, MS: multiple sclerosis, PNP: polyneuropathy, QNfL: neurofilament light chain ratio, S-NfL: serum neurofilament light chain

**Supplementary Table 2: Characteristics of MS and PNP patients**

|  | **MS (n=52)** | **PNP (n=21)** |
| --- | --- | --- |
| Sex, n (%)  - Male  - Female | 24 (46.1%)  18 (53.9%) | 13 (61.9%)  8 (38.1%) |
| Age at time point NfL (years), median (IQR) | 30 (24-36.8) | 55 (47.0-61.0) |
| Diagnosis, n (%)   - CIS - RRMS | 15 (28.8%)  37 (71.2%) | n.a. |
| Presence of CEL at time point of sample collection, n (%) | 17 (32.7%) | n.a. |

Abbreviations: CEL: contrast-enhancing lesions, CIS: clinically isolated syndrome, IQR: interquartile range, MS: multiple sclerosis, n.a.: not applicable, NfL: neurofilament light chain, RRMS: relapsing-remitting multiple sclerosis

## Supplementary Table 3: Spearman correlation of QAlb with S-NfL, CSF-NfL and QNfL

|  | **HC, n=41** | **IIH, n=65** | **MS, n=52** | **PNP, n=21** |
| --- | --- | --- | --- | --- |
| QAlb vs. S-NfL | r=0.05  p=0.74 | r=-0.05  p=0.72 | r=0.04  p=0.76 | r=-0.14  p=0.56 |
| QAlb vs. CSF-NfL | r=-0.08  p=0.65 | r=0.03  p=0.80 | r=0.23  p=0.11 | r=0.01  p=0.97 |
| QAlb vs. QNfL | r=-0.09  p=0.60 | r=0.10  p=0.41 | r=0.43  **p=0.002 | r=0.17  p=0.47 |

Abbreviations: QAlb: Albumin ratio, CSF-NfL: cerebrospinal fluid neurofilament light chain, HC: healthy controls, IIH: idiopathic intracranial hypertension, MS: multiple sclerosis, PNP: polyneuropathy, QNfL: neurofilament light chain ratio, S-NfL: serum neurofilament light chain

## Supplementary Table 4: QNfL derived from previously published CSF-NfL an P-NfL in patients with inflammatory polyneuropathies and controls (34)

|  | **GBS, n=41** | **CIDP, n=32** | **NIP, n=22** | **HC, n=39** |
| --- | --- | --- | --- | --- |
| log CSF-NfL, median | 3.4 | 3.1 | 2.9 | 2.6 |
| CSF-NfL (pg/ml), median | 2511.9 | 1258.9 | 794.3 | 398.1 |
| log P-NfL, median | 1.7 | 1.4 | 1.18 | 0.93 |
| P-NfL (pg/ml), median | 50.1 | 25.1 | 15.1 | 8.5 |
| QNfL, median | 50.1 | 50.1 | 52.5 | 46.8 |

Abbreviations: CIDP: chronic inflammatory demyelinating polyneuropathy, CSF-NfL: cerebrospinal fluid neurofilament light chain, GBS: Guillain-Barré syndrome, HC: healthy controls, NIP: non-inflammatory polyneuropathy, P-NfL: plasma neurofilament light chain, QNfL: neurofilament light chain ratio
